# The Occurrence, Pathways, and Risk Assessment of Heavy Metals in Raw Milk from Industrial Areas in China

**DOI:** 10.3390/toxics9120320

**Published:** 2021-11-26

**Authors:** Chuanyou Su, Yanan Gao, Xueyin Qu, Xuewei Zhou, Xue Yang, Shengnan Huang, Lei Han, Nan Zheng, Jiaqi Wang

**Affiliations:** 1 Milk and Dairy Product Inspection Center of Ministry of Agriculture and Rural Affairs, Institute of Animal Sciences, Chinese Academy of Agricultural Sciences, Beijing 100193, China; suchuanyou2010@hotmail.com (C.S.); gaoyanan2019@gmail.com (Y.G.); zhouxuewei@gdou.edu.cn (X.Z.); 82101182216@caas.cn (X.Y.); hsn1435007@hotmail.com (S.H.); lhan25122@gmail.com (L.H.); 2 College of Animal Science and Technology, Henan Agriculture University, Zhengzhou 450046, China; 3 Tianjin Mengde Group Co., Ltd., Tianjin 300400, China; quxueyin@hotmail.com

**Keywords:** heavy metals, raw milk, industrial, risk assessment, China

## Abstract

This study evaluated chromium (Cr), arsenic (As), cadmium (Cd), and lead (Pb) contamination in raw milk from industrial areas in China, identified the possible pathways of heavy metals from the environment to raw milk, and made a risk assessment of the consumption of heavy metals from milk consumption. The Cr, As, Cd, and Pb concentrations in raw milk, water and silage were analyzed using inductively coupled plasma mass spectrometry. The Cr and As in soil were analyzed by flame atomic absorption spectrometry and atomic fluorescence spectrometry, respectively. Cd and Pb in soil were determined by a Graphite furnace atomic absorption spectrophotometer. The Cr and As concentrations in milk from industrial areas were 2.41 ± 2.12 and 0.44 ± 0.31 μg/kg, respectively, which were significantly higher (*p* < 0.01) than those from non-industrial areas, which had levels of 1.10 ± 0.15 and 0.25 ± 0.09 μg/kg, respectively. Chromium was mainly transferred through the soil-silage-milk pathway, As was transferred through the water-silage-milk pathway, while Cd was mainly transferred through the soil (water)-silage-milk pathway. The contributions of each metal to the overall hazard index (HI) followed a descending order of As, Cr, Pb, and Cd, with values of 46.64%, 25.54%, 24.30%, and 3.52%, respectively. Children were at higher risk than adults.

## 1. Introduction

With increasing population, industrialization, and urbanization, various pollutants have been released into the environment. Some heavy metals have become widely distributed in the environment, in particular, chromium (Cr), arsenic (As), cadmium (Cd), and lead (Pb) [1], facilitating their entry in the human food chain [2]. Heavy metals have negative effects on both livestock health [3,4] and human health [5]. These metals are potentially toxic, causing hematologic, neurotoxic, and nephrotoxic effects even at low concentrations. Human exposure to these heavy metals has a negative effect on specific organs and may lead to metabolic disorders, fatigue, heart failure, and cancer [4,6,7,8]. This problem is particularly serious for children, whose immune systems are underdeveloped and for whom gastrointestinal absorption is not as efficient as in adults [9]. Hexavalent chromium (Cr VI), As, and Cd are group 1 carcinogens, while inorganic Pb is a group 2A carcinogen, as classified by the International Agency for Research on Cancer (IARC) [10]. In the United States, according to their occurrence, toxicity, and potential human exposure, As, Pb, Cd, and Cr were listed as the 1st, 2nd, 7th, and 17th priority contaminants in food by the Agency for Toxic Substances and Disease Registry (ATSDR) [11]. The levels of these heavy metals in milk can not only be used as a direct indicator of milk hygiene, but also as an indirect indicator of environmental pollution in the areas where raw milk is produced [1,12,13]. Thus, the monitoring of heavy metals in milk is vital for product quality and must be regarded as an essential public health procedure. In China, to protect human health, the government has set maximum level (ML) standards for Cr, As, and Pb in raw milk of 0.3, 0.1, and 0.05 mg/kg, respectively [14].

Sources of heavy metal pollution include mining, the proximity of roads, fuel combustion, and industrial areas, especially iron and steel plants [15]. In some areas, the concentrations of heavy metals in raw milk vary due to the differences in pollution levels [16,17]. Some researchers have focused on the assessment of potential health risks for residents in contaminated areas, including areas irrigated with wastewater [18] and areas in the vicinity of the petroleum extraction industry [4]. In China, only a few studies have attempted to assess the levels of heavy metals in milk, especially milk from cows reared in industrial areas. In our previous study, we conducted a risk assessment of heavy metals in raw milk from a variety of milk producing areas [19] and areas close to leather-processing plants [9]. However, few studies have assessed the risk of heavy metals to people (from infants to seniors) in areas with industrial sites such as cement production and power plants.

We hypothesized that Cr, As, Cd, and Pb levels in locally produced milk would be elevated if the levels were high in the local water, soil, and silage due to industrial activity. The aims of the study were to: (i) compare the heavy metal concentrations in raw milk from industrial and non-industrial areas in China; (ii) identify the relationships among heavy metals in raw milk, silage, water, and soil, and the possible pathways of heavy metals from the environment to raw milk; and (iii) evaluate the heavy metal exposure and health risks from milk consumption for local residents.

## 2. Materials and Methods

### 2.1. Study Area

During autumn 2019, samples were collected from 20 farms in Tangshan (5), Tianjin (7), Hohhot (6), Weifang (1), and Qiqihar (1) in China. Tangshan (117°31′–119°19′ E, 38°55′–40°28′ N; 7.72 million people) and Hohhot (110°46′–112°10′ E, 40°51′–41°8′ N; 3.45 million people) are industrialized cities with factories, including steel plants, cement plants, and waste incineration plants, which are all potential sources of environmental contamination. Tianjin (116°43′–118°4′ E, 38°34′–40°15′ N; 13.87 million people) is also a mega-city with many steel plants, cement plants, and a waste incineration plant. The farms selected industrial area were withen 30 km from the cement plants or waste incineration plant. Weifang (118°10′–120°01′ E, 35°41′–37°26′ N; 9.91 million people) and Qiqihar (122°24′–126°41′ E, 46°13′–48°56′ N; 5.59 million people) are cities located in agricultural areas and are close to a wetland reserve. Weifang and Qiqihar were therefore expected to be free from contamination and were considered to represent a control area.

### 2.2. Sampling

Raw milk was sampled from each farm from five lactating Holstein cows in their 3rd parity. A total of 100 milk samples were collected in polyethylene plastic bottles from 20 farms. Silage (1), drinking water (1), and soil samples (1) were also collected from each farm. Silage samples were randomly collected from the lower, middle, and upper parts of silos. Soil samples were taken from depths of 0–30 cm at six random points. Silage and soil samples were stored in polythene bags. The cows’ drinking water (groundwater) was sampled and stored in 200 mL plastic bottles. After being gathered, all the samples were stored at −20 °C.

### 2.3. Sample Preparation and Metal Analysis

Silage samples were dried in an oven at 65 °C and ground to a particle size of 1 mm. Raw milk (1 g) or silage (0.5 g) were added to a digestion vessel. Then, 5 mL 65% HNO_3_ (Suprapur, Merck, Darmstadt, Germany) was added, followed by 2 mL of 30% H_2_O_2_ (Suprapur^®^, Merck, Kenilworth, NJ, USA). After a 12 h predigestion, the mixture was digested by a microwave assisted reaction system (CEM Corporation, Matthews, NC, USA), according to a program reported by Zhou et al. (2017) [20]. Following digestion and cooling to room temperature, the digestate was diluted to 50 mL with ultrapure water. The liquid samples were analyzed by inductively coupled plasma mass spectrometry (ICP-MS) (Agilent 7700 Series ICP-MS, Agilent Technologies, Santa Clara, CA, USA). Water samples mixed with HNO_3_ (1% *v*/*v*) were also analyzed by ICP-MS.

The pretreatment and analysis of soil samples was conducted according to the method described by Zhou et al. (2019) [15]. Briefly, the samples were naturally air-dried and then ground in the laboratory. The Cr level in soil was measured by flame atomic absorption spectrometry (ICE 3500, Thermo Fisher Scientific, Waltham, MA, USA), and Cd and Pb levels were measured using a graphite furnace atomic absorption spectrophotometer (Z-2700, Hitachi, Tokyo, Japan) after digestion with HNO_3_, HCl, HClO_4_, and HF. The levels of As in soil were determined by atomic fluorescence spectrometry (AFS 9800, Beijing Kechuang Haiguang Instrument Company, Beijing, China) after digestion with HCl and HNO_3_.

The limits of detection (LODs) of Cr, As, Cd, and Pb in milk were 0.82, 0.028, 0.03, and 0.03 µg/kg, respectively. The LODs of Cr, As, Cd, and Pb in water and silage were, 0.130, 1.500, 0.003, and 0.015 µg/L and 0.04, 0.03, 0.40, and 2.40 µg/kg, respectively. The LODs of Cd, As, Cr, and Pb in soil were 0.01, 0.02, 5, and 0.1 mg/kg, respectively. Values of heavy metals which under the LOD were calculated by a constant value of half the LOD [17].

### 2.4. Risk Assessment

#### 2.4.1. Exposure Assessment

An exposure assessment was conducted for local residents (aged 3 to 69 years) using the mean Cr, As, Cd, and Pb levels in milk from the industrial areas and the corresponding recommended milk consumption rates [21] and bodyweight [22]. The metal exposure from milk consumption was determined as follows [23]:

EDI = C × DI/BW,(1)

where EDI is the estimated daily intake, BW is the body weight (kg), C is the milk heavy metal concentration (μg/kg), and DI is the daily milk intake (kg).

#### 2.4.2. Target Hazard Quotient (THQ)

The non-carcinogenic risk potential chronic risk of heavy metals was expressed as the THQ and as a hazard index (HI). It was calculated as follows:

THQ = EDI/RfD,(2)

where RfD is the oral reference dose, with values of 3 × 10^−3^, 3 × 10^−4^, 1 × 10^−3^, and 4 × 10^−3^ mg/kg/d for Cr, As, Cd, and Pb, respectively [24,25,26,27,28].

#### 2.4.3. Hazard Index

The HI was used to assess the chronic risk from multiple heavy metals. It was calculated according to Equation (3). There was no risk to human health if HI < 1 [18,29].

HI = ∑THQ,(3)

### 2.5. Data Analysis

The data were analyzed with SPSS (IBM, Endicott, NY, USA) version 20. Results were expressed as a mean ± standard deviation (SD). As the data were non-normal distribution, heavy metal contents in raw milk from non-industrial and industrial areas were compared using non-parametric Kruskal-Wallis tests. Differences were considered to be statistically significant at *p* < 0.05. To explore these relationships, a Spearman’s correlation analysis of the relationships among heavy metals in milk and silage, water, and soil was conducted.

## 3. Results and Discussion

### 3.1. Levels of Cr, As, Cd, and Pb in Raw Milk

**Figure 1:**
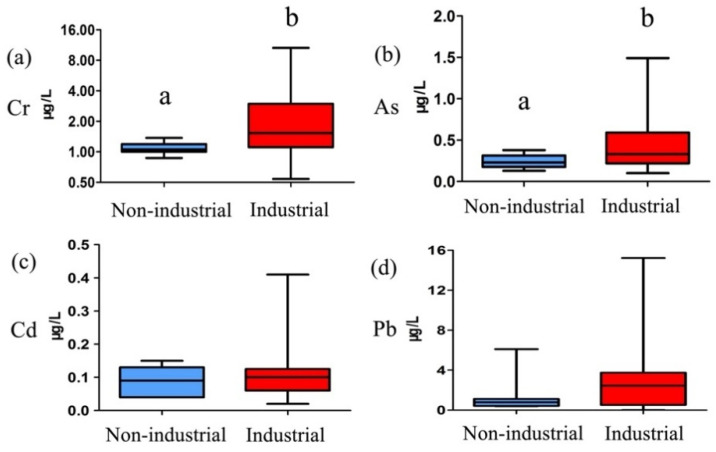
Comparison of heavy metal concentrations in raw milk from non-industrial and industrial areas. (**a**) Comparsion of Cr concentrations in raw milk form non-industrial and industrial areas; (**b**) Comparsion of As concentrations in raw milk form non-industrial and industrial areas; (**c**) Comparsion of Cd concentrations in raw milk form non-industrial and industrial areas; (**d**) Comparsion of Cd concentrations in raw milk form non-industrial and industrial areas. Superscript lower-case letters (a, b) different in the same subfigures indicate significant differences (*p* < 0.05).

Concerns regarding the effects of heavy metals on human health have arisen due to their accumulation in the environment, particularly in livestock and agricultural production, increasing their potential to leave residue in human food [4]. Heavy metal exposure is positively related to the development of various diseases. Data collection and continuous monitoring are required to fully evaluate the impacts of heavy metals in food on human health in future studies [16]. As one potential source of heavy metals in the human diet, the monitoring of milk is therefore considered necessary [30]. In this study, we evaluated the heavy metal concentrations in raw milk from industrial and non-industrial areas in China (Table 1 and Figure 1). The ranges of the Cr, As, Cd, and Pb levels in milk were 0.54–10.61, 0.10–1.49, 0.02–0.39, and ND–15.22 μg/kg, respectively. The Cr, As, and Pb concentrations were below the maximum levels (MLs) set in China of 300, 100, and 50 μg/kg, respectively [14]. The Cd concentration was below the limit of the Codex Alimentarius Commission (CAC) (20 μg/kg) [31]. The highest Cr, As, and Cd concentrations in raw milk samples were observed in Hohhot. The highest Pb concentration in raw milk was observed in Tianjin, which was located in an industrial area.

The Cr and As levels in milk from industrial areas were 2.41 ± 2.12 and 0.44 ± 0.31 μg/kg, respectively, which were significantly higher (*p* < 0.01) than those from non-industrial areas, in which the levels were 1.10 ± 0.15 and 0.25 ± 0.09 μg/kg, respectively (Figure 1). Previous studies have shown that industrial activity can lead to high heavy metal levels in milk [4,13]. One previous study reported that levels of As and Pb in milk from cows reared in regions with a high traffic intensity and industrial activity were significantly (*p* < 0.05) higher than those from a rural area [32].

The heavy metal concentrations in raw milk reported by previous studies are summarized in Table 2. The milk Cr levels (0.54–10.61 μg/kg) measured in this study were consistent with levels reported elsewhere in China [15], as well as in Romania [33] and Spain [34], but were lower than the Cr concentrations reported in Bangladesh [35], Pakistan [29], and Turkey [36]. The range of As levels (0.10–1.49 μg/kg) in raw milk were consistent with the ranges observed in Korea [28], Romania [33], and elsewhere in China [15,17]. The As levels in this study were lower than the concentrations reported in Mexico [37] and Turkey [36,38]. The Cd concentrations in milk (0.02–0.39 μg/kg) measured in this study were consistent with levels previously reported in China [15,17], northeast Iran [39], western Iran [40], and Spain [34], but were lower than the Cd concentrations reported in Egypt [41], Pakistan [29], and Peru [42]. The Pb concentrations in milk (0–15.22 μg/kg) measured in this study were consistent with levels reported elsewhere in China [15,17], as well as in Iran [43], Korea [28], northeast Iran [39], Pakistan [44], Romania [33], and Spain [34], but were lower than those reported in Egypt [41], Peru [42], Turkey [32,36], and western Iran [40].

### 3.2. Levels of Cr, As, Cd, and Pb in Silage, Water, and Soil

Anthropogenic activities, especially industrial activities, will increase heavy metal accumulation in the environment. In industrial areas, heavy metal pollution in silage is usually more severe, resulting in elevated levels in blood, which are then excreted into milk [48]. For animals, polluted drinking water is also considered to be an important source of heavy metal exposure [49]. Therefore, silage and water have been monitored to estimate the source of heavy metal contamination [29,50]. Heavy metal levels in silage, water, and soil from industrial and non-industrial areas were shown in Table 3. The Cr, As, Cd, and Pb concentrations in silage from industrial and non-industrial areas were in the ranges of 1.57–4.84, 0.06–0.29, 0.01–0.09, and 0.26–1.17 mg/kg and 1.05–1.66 0.05–0.11 0.02–0.03, and 0.16–0.22 mg/kg, respectively. The ranges of Cr, As, Cd, and Pb concentrations in water from industrial and non-industrial areas were ND–4.93, 0.15–2.80, ND–0.040, and 0.01–0.10 μg/L and 0.07–0.38, 0.30–0.47, 0.001–0.005, and 0.09–0.15 μg/L, respectively. The ranges of the four heavy metals in soil from industrial and non-industrial areas were 42–218, 3.73–17.30, 0.06–0.27, and 21–42 mg/kg, and 59–133, 5.48–10.20, 0.09–0.10, and 24–29 mg/kg, respectively. Silage Cr, As, Cd, and Pb levels were below the Chinese MLs (5, 2, 1, and 30 mg/kg, respectively) [51]. The Cr, As, Cd, and Pb concentrations in water and soil were lower than the MLs for drinking water (50, 10, 5, and 10 μg/L, respectively) [52] and the risk screening value for agricultural soil (250, 240, 0.8, and 170 mg/kg, respectively) [53].

### 3.3. The Relationships among Heavy Metals in Milk, Silage, Water, and Soil

Previous studies have found that heavy metals in milk are related to the environmental conditions. Heavy metal pollution in the environment can contaminate milk through feed and water. The relationships between heavy metals in milk and silage, water, and soil were shown in Table 4.

A strong correlation between the Cr (r = 0.555) and Cd (r = 0.709) concentrations in milk and forage was reported previously [54]. In the present study, the Cr levels in milk and silage were moderately positively correlated (r = 0.626); however, the Cr concentrations in milk had almost no correlation with those in drinking water (r = 0.041), and a weak negative correlation with the soil concentration (r = 0.344). The correlation coefficient between soil and milk Cr was previously reported to be 0.0007, while for the relationship between the forage and milk concentrations it was 0.068 [55]. The As concentrations in milk and water were moderately positively correlated (r = 0.637), while the milk concentrations were weakly positively correlated with silage concentrations (r = 0.326). The As level between milk and soil displayed a weak negative correlation (r = −0.112). In a previous study, a positive correlation was observed between the As concentrations in milk and the drinking water provided for cattle on farms, with Pearson correlation coefficients in the range of 0.926–0.974 [56]. The As concentrations in milk and water were weakly positively correlated (r = 0.37), but had almost no correlation with the soil concentration (r = −0.03) [15].

The milk Cd concentration was moderately positively correlated with the silage (r = 0.676) and soil (r = 0.557) concentrations. A weakly positive correlation was observed between the Cd concentrations in milk and water samples (r = 0.119). Fozia Batool et al. [54] investigated the possible pathways by which Cd was transferred to milk. The cows’ fodder and the soil on which the fodder was grown were analyzed. There was a strong correlation for Cd (r = 0.709). The Pb concentration in milk had weak positive correlations with the concentrations in silage (r = 0.194) and soil (r = 0.327), but almost no correlation (r = 0.046) with the concentration in water in the present study. Similarly, a weak correlation for Pb was found in the same media by Fozia Batool et al. [54]. Zhou et al. [15] investigated the relationships between Cd and Pb in milk samples and the heavy metal concentrations in silage, water, and soil. A moderate positive correlation between the milk and soil concentrations was found for Cd (r = 0.65); however, there was a negative correlation between the milk and water concentrations (r = −0.75).

The Cr concentrations in silage were moderately positively correlated with soil concentrations (r = 0.556), and the As concentrations in silage were moderately positively correlated with water concentrations (r = 0.556). The Cd and Pb concentrations in silage were moderately positively correlated with soil concentrations (r = 0.739, r = 0.654) and weakly positively correlated with water concentrations (r = 0.444, r = 0.424).

Based on the relationships among the heavy metals in milk, silage, water, and soil, it was determined that Cr was mainly transferred through the soil-silage-milk pathway, As was mainly transferred through the water-silage-milk or water-milk pathways, and Cd were mainly transferred through the soil (water)-silage-milk pathway in the present study. Pb in raw milk may come primarily from other feed materials.

### 3.4. Exposure Assessment for Cr, As, Cd, and Pb from Milk Consumption

Dietary exposure is an effective method to assess food pollutants and to determine the potential health risks [57]. The EDI values of Cr, As, Cd, and Pb were calculated for people of various ages (3–69 years), as shown in Table S1. The Cr, As, Cd, and Pb results for women and men were 0.0120–0.0758, 0.0022–0.0138, 0.0005–0.0035, and 0.0151–0.0959 μg/kg/day and 0.0101–0.0726, 0.0018–0.0132, 0.005–0.0033, and 0.0128–0.0919 μg/kg/day, respectively. The EDI values for these metals were lower than the RfD values of Cr, As, Cd, and Pb (3.0, 0.3, 1, and 4 μg/kg/day, respectively).

**Figure 2:**
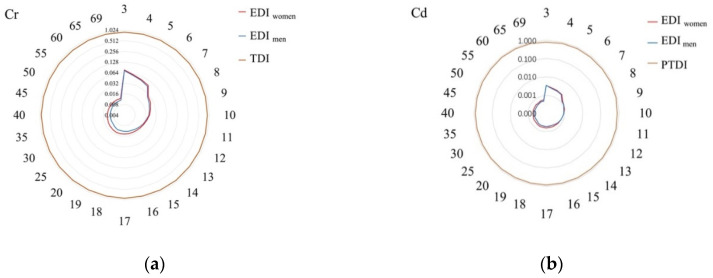
Comparison of the Cr and Cd exposure values (μg/kg/day) with TDI and PTDI for people aged 3 to 69 years. (**a**) Comparison of the Cr exposure values (μg/kg/day) with TDI for people aged 3 to 69 years; (**b**) Comparison of the Cd exposure values (μg/kg/day) with PTDI for people aged 3 to 69 years.

The tolerable daily intake (TDI) for oral Cr (VI) exposure of 0.9 μg/kg BW/d was established by the International Programme on Chemical Safety (ICPS) [58]. In 2010, a provisional tolerable monthly intake (PTMI) of 25 μg/kg BW (equivalent 0.83 μg/kg BW/d for PTDI) for Cd was established by the Joint FAO-WHO Expert Committee Report on Food Additives (JEFCA) [59]. Due to the tolerable weekly intake of inorganic As [59] and Pb [59] being withdrawn by JECFA, only the Cr and Cd exposure values were compared with the TDI and PTDI, respectively (Figure 2). The EDI value of Cr for milk samples was far below the TDI (0.9 μg/kg BW). The EDI value of Cd was below 0.83 μg/kg BW/d for people aged 3 to 69, indicating that there was no health risk associated with the Cr and Cd intake from milk consumption.

Due to the absence of an As and Pb PTWI value, a non-carcinogenic risk assessment model was used to evaluate the potential risk of these heavy metals through milk for the local residents. The THQ for Cr, As, Cd, and Pb was based on their mean concentrations in industrial areas [21].

**Figure 3:**
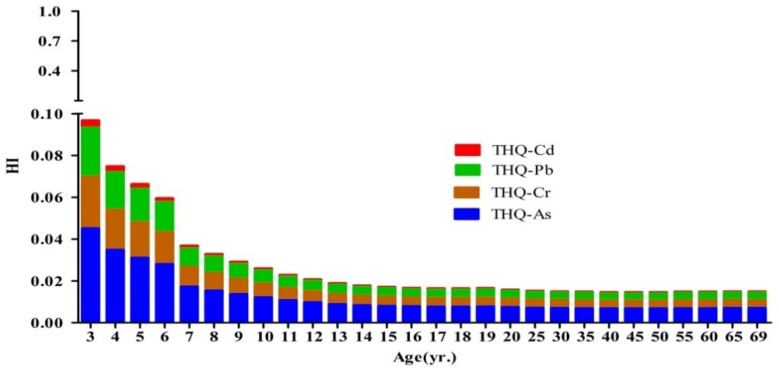
Target hazard quotient (THQ) and hazard index (HI) for four heavy metals exposure following milk consumption for individuals from 3 to 69 years of age.

The values of THQ and the HI for milk ingestion by local residents are summarized in Figure 3. There was an inverse relationship between the THQ values of Cr, As, Cd, and Pb with the age of exposure. The THQ values followed a descending order of As, Pb, Cr, and Cd. The contributions of As, Cr, Pb, and Cd to the total THQ were 46.64%, 25.54%, 24.30%, and 3.52%, respectively. As shown from the data, the THQ values never exceeded the HI threshold of 1.

The greatest risk was presented to young children due them having the lowest BW and largest milk intake of the population groups studied. On a BW basis, children could have an exposure more than twice as high as adults [59]. In terms of their THQ, men had a lower risk than women of the same age. For the HI value, As made the largest contribution, followed by Pb, Cr, and Cd. Previous research has shown that As had the largest THQ value among the four metals investigated [9,18,60], which was supported by the results of the present study. The HI values for people aged 3 to 69 years were in the range of 0.0145–0.0967, i.e., far less than the threshold of 1. This indicates that the exposure level of these heavy metals through milk consumption will not cause adverse effects over a lifetime. It should be recognized that milk is not the single food item consumed by humans. Milk and dairy products represent only a small proportion (16.5–24.5%) of the total mass of food consumed per day in China [21]. The non-carcinogenic risk presented by heavy metals could be higher when other foods are considered.

## 4. Conclusions

The Cr and As levels in milk from cows reared in industrial areas were significantly higher than the levels from non-industrial areas. Chromium was mainly transferred through the soil-silage-milk pathway, As was mainly transferred through the water-milk or water-silage-milk pathways, and Cd was mainly transferred through the soil (water)-silage-milk pathway. Children were at higher risk than adults.

## Figures and Tables

**Table 1:** The levels of heavy metals (μg/kg) in raw milk from five areas in China (*n* = 100).

Area		N		Cr	As	Cd	Pb
Non-industrial	I	5	Mean ± SD	1.17 ± 0.15 ^bc^	0.33 ± 0.03 ^c^	0.05 ± 0.02 ^c^	0.45 ± 0.07 ^b^
			Range	1.06–1.37	0.31–0.38	0.04–0.09	0.40–0.56
	II	5	Mean ± SD	1.04 ± 0.15 ^c^	0.17 ± 0.02 ^b^	0.11 ± 0.04 ^ab^	0.94 ± 0.19 ^b^
			Range	0.87–1.28	0.13–0.19	0.04–0.15	0.74 –1.18
Industrial	III	25	Mean ± SD	1.18 ± 0.22 ^c^	0.24 ± 0.09 ^bc^	0.13 ± 0.09 ^a^	4.46 ± 2.45 ^a^
			Range	0.90–1.89	0.12–0.53	0.04–0.41	2.26 –12.68
	IV	35	Mean ± SD	1.86 ± 1.24 ^b^	0.42 ± 0.24 ^a^	0.11 ± 0.06 ^a^	2.68 ± 3.60 ^b^
			Range	0.54–7.03	0.16–1.47	0.02–0.31	ND–15.22
	V	30	Mean ± SD	4.09 ± 2.72 ^a^	0.64 ± 0.38 ^a^	0.09 ± 0.08 ^bc^	2.29 ± 3.10 ^b^
			Range	0.87–10.61	0.10–1.49	0.02–0.39	ND–10.12
Total		100	Mean ± SD	2.28 ± 2.01	0.42 ± 0.40	0.10 ± 0.07	2.18 ± 3.16
			Range	0.54–10.61	0.10–1.49	0.02–0.39	ND–15.22
Maximum level				300	100	20	50

Note: N represents the number of samples; I, II, III, IV, and V represent the areas of Qiqihar, Weifang, Tangshan, Tianjin, and Hohhot, respectively; Superscript lower-case letters (a, b, c) different in the same column indicate significant differences (*p* < 0.05).

**Table 2:** Heavy metal concentrations in raw milk reported from different countries (μg/kg).

Country	Year	N	Cr	As	Cd	Pb	Reference
Argentina	—	52	—	—	1.47 (0–17.0)	—	[45]
Bangladesh	2014–2015	30	373 ± 8 (BDL–1233)	—	24 ± 9 (BDL–73)	33 ± 6 (BDL–200)	[35]
Croatia	2010–2014	249	—	—	—	11.4 ± 8.08 (5.11–131)	[46]
China	2016	997	—	0.31 ± 1.02 (0.05–15.77)	0.05 ± 0.07 (0.001–0.69)	1.75 ± 3.73 (0.14–38.61)	[17]
China	2017	60	0.87 ± 1.02 (0.02–5.01)	0.06 ± 0.20 (0.0002–1.53)	0.09 ± 0.006 (0.01–0.27)	1.22 ± 1.62 (0.03–10.46)	[15]
Egypt	—	20	—	—	51 ± 5	214 ± 21	[41]
Iran	2014	32	—	15.2–25.9	—	—	[47]
Iran	—	118	—	—	3.47 (ND–100)	38.42 (ND–250)	[4]
North-east Iran	—	720	—	—	0.3 ± 0.3	12.9 ± 6.1	[39]
Western Iran	2014	36	—	—	0.036 ± 0.28 (0.06–0.78)	32.8 ± 20.80 (15.7–68)	[40]
Korea	2012	33	—	1.90 ± 0.068	2.38 ± 0.023	(3.35 ± 0.076)	[28]
Mexico	—	60	30 ± 10	120 ± 80	—	30 ± 10	[37]
Pakistan	2014	480	—	—	1	14	[44]
Pakistan	2010–2011	30	251.7 ± 32 (190–303)	—	20 ± 4 (14–31)	—	[29]
Peru	2018	20	—	—	19.7 ± 7.3 (11.0–32.0)	580 ± 18 (540–600)	[42]
Romania	—	—	4.56	1.02	1.09	6.57	[33]
Spain	—	347	4.03 ± 3.43 (<LOD–24.35)	—	0.40 ± 0.28 (<LOD–1.73)	2.85 ± 1.091 (0.55–18.7)	[34]
Turkey	—	20	(70–1227)	(103–3326)	(0.1–4)	(25–124)	[36]
Turkey	—	20	—	—	—	40.5 (23–58)	[32]
Turkey	2015–2016	112	—	17.5 ± 1.7 (ND–42.8)	—	—	[38]
China	2019	120	(0.54–10.61)	(0.10–1.49)	(0.02–0.39)	(ND–15.22)	This study

Note: — Not mentioned in the reference.

**Table 3:** Heavy metal levels in silage, water, and soil from industrial and non-industrial areas.

Samples	Heavy Metals	Non-Industrial	Industrial	Maximum Level
Silage (mg/kg)	Cr	1.05–1.66	1.57–4.84	5
	As	0.05–0.11	0.06–0.29	2
	Cd	0.02–0.03	0.01–0.09	1
	Pb	0.16–0.22	0.26–1.17	30
Water (μg/L)	Cr	0.07–0.38	ND–4.93	50
	As	0.30–0.47	0.15–2.80	10
	Cd	0.001–0.005	ND–0.040	5
	Pb	0.09–0.15	0.01–0.10	10
Soil (mg/kg)	Cr	59–133	42–218	250 ^a^
	As	5.48–10.20	3.73–17.30	240 ^a^
	Cd	0.09–0.10	0.06–0.27	0.8 ^a^
	Pb	24–29	21–42	170 ^a^

Note: ^a^ Risk screening vale for soil of agricultural land and when pH > 7.5.

**Table 4:** Relationships between heavy metals in milk, silage, water, and soil.

Samples	Cr	As	Cd	Pb
Milk-silage	0.626 **	0.326	0.676 **	0.194
Milk-water	0.041	0.637 **	0.119	0.046
Milk-soil	0.344	−0.112	0.557 *	0.327
Silage-water	0.076	0.556 *	0.444 *	0.424
Silage-soil	0.604 **	0.180	0.739 **	0.654 **
Water-soil	−0.045	0.113	0.237	0.341

Note: * *p* < 0.05, ** *p* < 0.01.

## Data Availability

Data is contained within the article or supplementary material.

## Supplementary Materials

The following are available online at https://www.mdpi.com/article/10.3390/toxics9120320/s1, Table S1: The EDI values of Cr, As, Cd, and Pb following milk consumption by people of different ages.
