# Supplementary material for: The Occurrence, Pathways, and Risk Assessment of Heavy Metals in Raw Milk from Industrial Areas in China
Source: Toxics. 2021 Nov 26;9(12):320. doi: 10.3390/toxics9120320 (PMC8708092; doi:10.3390/toxics9120320)
Supplement: Supplementary file 1 [file toxics-09-00320-s001.zip › toxics-1457849-supplementary.pdf]

toxics

Supplementary Materials: The Occurrence, Pathways, and Risk Assessment of Heavy Metals in Raw Milk from Industrial Areas in China

Chuanyou Su, Yanan Gao, Xueyin Qu, Xuewei Zhou, Xue Yang, Shengnan Huang, Lei Han, Nan Zheng and Jiaqi Wang

Table S1. The EDI values of Cr, As, Cd, and Pb following milk consumption by people of different ages.

Age (yr.)	Cr		As		Cd		Pb	
	Woman	Man	Woman	Man	Woman	Man	Woman	Man
3	0.0758	0.0726	0.0138	0.0133	0.0035	0.0033	0.0959	0.0919
4	0.0585	0.0560	0.0107	0.0102	0.0027	0.0026	0.0741	0.0708
5	0.0523	0.0497	0.0095	0.0091	0.0024	0.0023	0.0661	0.0629
6	0.0474	0.0445	0.0087	0.0081	0.0022	0.0020	0.0600	0.0564
7	0.0293	0.0272	0.0053	0.0050	0.0013	0.0012	0.0370	0.0344
8	0.0262	0.0242	0.0048	0.0044	0.0012	0.0011	0.0332	0.0306
9	0.0231	0.0215	0.0042	0.0039	0.0011	0.0010	0.0292	0.0272
10	0.0204	0.0194	0.0037	0.0035	0.0009	0.0009	0.0258	0.0246
11	0.0178	0.0173	0.0033	0.0032	0.0008	0.0008	0.0225	0.0218
12	0.0162	0.0155	0.0030	0.0028	0.0007	0.0007	0.0206	0.0196
13	0.0151	0.0139	0.0028	0.0025	0.0007	0.0006	0.0191	0.0176
14	0.0143	0.0129	0.0026	0.0023	0.0007	0.0006	0.0182	0.0163
15	0.0140	0.0122	0.0026	0.0022	0.0006	0.0006	0.0177	0.0154
16	0.0137	0.0118	0.0025	0.0021	0.0006	0.0005	0.0174	0.0149
17	0.0136	0.0114	0.0025	0.0021	0.0006	0.0005	0.0173	0.0145
18	0.0137	0.0114	0.0025	0.0021	0.0006	0.0005	0.0174	0.0144
19	0.0138	0.0114	0.0025	0.0021	0.0006	0.0005	0.0175	0.0144
20	0.0134	0.0108	0.0025	0.0020	0.0006	0.0005	0.0170	0.0136
25	0.0131	0.0103	0.0024	0.0019	0.0006	0.0005	0.0165	0.0130
30	0.0127	0.0101	0.0023	0.0018	0.0006	0.0005	0.0161	0.0128
35	0.0125	0.0101	0.0023	0.0018	0.0006	0.0005	0.0158	0.0128
40	0.0123	0.0102	0.0022	0.0019	0.0006	0.0005	0.0155	0.0129
45	0.0121	0.0102	0.0022	0.0019	0.0006	0.0005	0.0153	0.0129
50	0.0120	0.0102	0.0022	0.0019	0.0005	0.0005	0.0151	0.0130
55	0.0121	0.0105	0.0022	0.0019	0.0006	0.0005	0.0154	0.0132
60	0.0121	0.0107	0.0022	0.0020	0.0006	0.0005	0.0153	0.0135
65	0.0122	0.0109	0.0022	0.0020	0.0006	0.0005	0.0155	0.0137
69	0.0122	0.0109	0.0022	0.0020	0.0006	0.0005	0.0155	0.0137
